# Using Patient Health Profile Evaluation for Predicting the Likelihood of Retinopathy in Patients with Type 2 Diabetes: A Cross-Sectional Study Using Latent Profile Analysis

**DOI:** 10.3390/ijerph19106084

**Published:** 2022-05-17

**Authors:** Shang-Jyh Chiou, Kuomeng Liao, Kuan-Chia Lin, Wender Lin

**Affiliations:** 1 Department of Health Care Management, National Taipei University of Nursing and Health Sciences, Taipei 112303, Taiwan; 2 Department of Endocrinology and Metabolism, Zhongxiao Branch, Taipei City Hospital, Taipei 115006, Taiwan; dah67@tpech.gov.tw; 3 Institute of Hospital and Health Care Administration, National Yang-Ming University, Taipei 112213, Taiwan; kuanchia@nycu.edu.tw; 4 Department of Health Care Administration, Chang Jung Christian University, 1 Changda Rd., Gueiren District, Tainan City 711301, Taiwan; vincelin@mail.cjcu.edu.tw

**Keywords:** patient health profiles, diabetic retinopathy, self-efficiency, self-management, latent profile analysis, type 2 diabetes

## Abstract

**Background:** To determine whether long-term self-management among patients with type 2 diabetes mellitus has the risk of developing complications. **Methods:** We conducted a survey of self-management behavior using diabetes self-management scales (DMSES-C and TSRQ-d) from November 2019 to May 2020 linked with biomarkers (glucose, lipid profile, blood pressure, and kidney function), and the varying measure values were transformed into normal rate proportions. We performed latent profile analysis (LPA) to categorize the patient into different patient health profiles using five classes (C1–C5), and we predicted the risk of retinopathy after adjusting for covariates. **Results:** The patients in C1, C2, and C4 had a higher likelihood of retinopathy events than those in C5, with odds ratios (ORs) of 1.655, 2.168, and 1.788, respectively (*p* = 0.032). In addition, a longer duration of diabetes was correlated with an increased risk of retinopathy events as well as being elderly. **Conclusions:** Optimal biomarker health profiles and patients with strong motivation pertaining to their T2DM care yielded better outcomes. Health profiles portraying patient control of diabetes over the long term can categorize patients with T2DM into different behavior groups. Customizing diabetes care information into different health profiles raises awareness of control strategies for caregivers and patients.

## 1. Introduction

The International Diabetes Federation published a global report on diabetes in 2019 and showed that the number of diabetic patients increased from 108 million in 1980 to 463 million in 2019, and most of these patients were diagnosed with type 2 diabetes mellitus (T2DM) [1]. This increased prevalence has had an enormous impact on health-care systems due to the disease burden and cost of management [2]. Diabetes is also a leading cause of death globally and is associated with the consumption of more medical resources due to the aging society. Nonetheless, T2DM is preventable and controllable when patients follow the guidelines related to medication, diet, exercise, and blood glucose monitoring and when patients attend annual eye and foot checkups. Moreover, insulin is a key player in developing type 2 diabetes, especially in severe situations. Physical activity makes diabetes patients more sensitive to insulin as well as weight loss (with diet control) [3,4]. These lifestyle changes are the cornerstone of diabetes management and really work. Most studies have indicated that patients with diabetes can have a good quality of life with a low risk of complications [5] if they can maintain their diabetes status at an optimal level long term; such effort is driven by the persistence of the patients and requires self-control behavior. Hence, the American Diabetes Association (ADA) still emphasizes the importance of self-management in diabetes care [6].

For the management of diabetes, the cooperation between patients and medical teams not only leads to optimal glycemic levels in patients but can also reduce the risk of complications in the future [7]. Successful disease management relies on optimal and acceptable patient behavior in diabetes care; however, patient behavior is always a complex issue. Some patients, for example, work hard to maintain a proper diet and adhere to an exercise routine but have little improvement in reducing their HbA1c levels, whereas some patients adopt less strict lifestyles and still have acceptable HbA1c levels. Nonetheless, studies have confirmed the long-term impact of failing to maintain a healthy lifestyle, including diet and exercise, on the risk of developing complications such as diabetic retinopathy (DR), diabetic foot and renal disease requiring dialysis [8,9].

DR is the leading cause of vision loss in patients with chronic diabetes and in those patients with poorly managed blood glucose levels [10]. The worldwide prevalence of DR among patients with diabetes is approximately 27% [11], and patients with chronic diabetes have an increased risk of DR. Controlling diabetes and blood pressure and maintaining the patient’s HbA1c levels between 6% and 7% could reduce the risk of developing DR [12]. In diabetes research, most studies have usually used the HbA1c levels as an indicator to determine whether diabetes is under control, and this index is commonly used to predict the risk of complications [13]. Numerous studies have focused on the use of medications in diabetic therapy and care, but research examining the importance of healthy behaviors in patients is limited. Therefore, conducting detailed research that would provide more information and evidence to support the conceptualization of useful health policy strategies is crucial to improve diabetes care.

Regarding patient behavior, self-efficacy and self-management are essential factors in diabetes-related health behaviors [14,15,16] and can reduce the risk of diabetes-related adverse effects. For example, weight control can reduce the risk of death [17], a Mediterranean diet can reduce the risk of cardiovascular disease [18], and better self-control behaviors, along with improved health cognition, can improve the long-term diabetes control [19]. However, few studies have investigated multiple factors, including patient behaviors with multiple biomarkers, together in diabetes control to uncover the relationship between patient behavior combined with the biomarkers in patient health profiles and the risk of complications. We are particularly interested in whether different patient health profiles will result in different complication risk levels in the future.

The multiple dimensions of diabetes control and its many variables introduces challenges in the analysis and clarification of correlations. To address these challenges in traditional research, we adopted latent profile analysis (LPA) to test the hypothesis that self-management assessment and biomarker information in different patient health profiles have different effects on the risk of developing complications. A participant-oriented approach can provide rich and concise information and can determine the patient’s effort in diabetes control over the study period. In addition, after considering the possible biomarkers that may be associated with the disease control situation, we used a fresh concept with varied biomarker characteristics (e.g., serum HbA1c, kidney function, and lipidomic profile changes) in the study period by transforming the varied measure values into the normal rate proportion to further evaluate diabetes control. Nonetheless, it is worth trying to use patient health profiles, including a self-management assessment with normal biomarker rates, to help patients understand the risk of complications with the different diabetes control efforts.

The ability to prolong life encourages patients toward self-management and compliance with the clinical guidelines, which is essential in the management of chronic diseases such as diabetes. Thus, the purpose of this study was to conduct a survey linking biomarker information analyzed by LPA to structure the patient behavior based on different patient health profiles and to predict the risk of complications (diabetic retinopathy) in the future. Using the concept of patient health profiles can help clinical practitioners to be aware of the patients’ behavior in their diabetes care. Our results will help health authorities provide incentives that encourage improvements in patient behavior and help clinical providers to conceive of useful strategies to support the patients’ adherence to diabetes care for better health outcomes.

## 2. Materials and Methods

### 2.1. Study Design, Setting and Participants

This is a cross-sectional study that collected self-management evaluations of diabetes control from a patient survey and linked 7-year multi-biomarker records from a health information system (HIS) to investigate the likelihood of a DR event. We enrolled T2DM patients from the Department of Metabolism of a regional hospital in the Northern part of Taipei, where the patients received one-stop services related to diabetes care in the hospital. This study was conducted from November 2019 to May 2020, and the data were collected from 601 subjects during the initial recruitment. After the data collection procedure, 570 patients remained in this study, of whom 31 were excluded because of missing identification (ID; *n* = 1) or double ID (*n* = 30). Before the survey, all patients voluntarily participated and were required to provide written consent, which included agreeing to include their biomarker data from the hospital’s HIS in this study for analysis. After the survey, we provided the patients with NTD 100 gift cards as participant compensation.

### 2.2. Dependent Variable

The hospital’s HIS provided the medical records from 2013 to 2019 for the patients who completed the survey and who agreed to participate in the study. Therefore, we defined a retinopathy event from the medical codes (indirect ophthalmoscopy for either the right or left eye) indicated in the hospital’s HIS. Any abnormal indirect ophthalmoscopy result was defined as a retinopathy event (Yes/No).

### 2.3. The Questionnaires

The instruments used in this study included personal information such as age, sex, education, diabetes onset date, and variables associated with diabetes care, including the self-reported comprehensive level of health education received from medical staff (1–10, with 10 representing the highest educational level); the Diabetes Management Self-efficacy Scale in Chinese (DMSES-C); the Treatment Self-regulation Questionnaire in Diabetes (TSRQd), a self-report assessment regarding the self-management of diabetes control with respect to medication, healthy diet, glucose monitoring, and regular exercise (1–5, with 5 being the highest score); and one item related to health status. Notably, the original DMSES and TSRQd have 20 and 19 items, respectively. The two questionnaires were translated into Chinese, with an acceptable validity and reliability [20,21,22]. After consulting with the team in charge of translations and the experts who had experience using the DMSES-C and TSRQd, our team used the shorter version, which excluded some items in the two questionnaires. Thus, in this study, we used the DMSES-C questionnaire, which included only 11 items, and the TSRQd, which included 15 items. We performed a confirmatory factor analysis to assess the validity of these questionnaires. The results shown in the supplemental file (Tables S1 and S2) confirm that the 11 items of the DMSES-C questionnaire and the 15 items of the TSRQd were acceptable. In addition, the TSRQd could be categorized into two dimensions from the original design: autonomous (A) and controlled regulations (C). Then, we used TSRQd_A and TSRQd_C to define them in this study. Finally, regarding the health status questions in the survey, we asked the patients the following question: “Compared with the previous 12 months, how would you evaluate your current health status (better, neutral, or worse)?”.

### 2.4. Linking to Biomarkers

As mentioned earlier, we obtained biomarker information from the HIS, including the blood glucose (HbA1C) level, lipid profile (low-density lipoprotein (LDL), high-density lipoprotein (HDL), and triglyceride (TG) levels), blood pressure (systolic blood pressure (SBP)), and kidney function (creatinine and albumin-to-creatinine ratio (ACR)). Each patient had multiple results of the seven biomarkers from 2013 to 2019. Furthermore, we calculated the normal rate of the seven biomarkers on the basis of the standards: HbA1c levels < 7.0%, LDL levels ≤ 100 mg/dL, HDL levels ≥ 40 mg/dL (male), HDL levels ≥ 50 mg/dL (female), TG levels < 150 mg/dL, SBP ≤ 120 mmHg, creatinine levels of 0.7–1.5 mg/dL (male) and 0.5–1.2 mg/dL (female), and ACR < 30 mg/mmol. For example, if one subject had HbA1c results of 7.1(+), 7(+), 6.9(–), 7.1(+), and 6.7(−), then, the normal rate was 2/5 = 0.4. This was done for all biomarkers to represent the diabetes control status over the study period. The normal rate is 0–1, and higher values indicate better control in diabetes care when based on the biomarker information. Moreover, the LPA model was used to classify the normal rate values of the seven biomarkers with the self-management assessment from the questionnaire.

### 2.5. LPA Results

**Figure 1:**
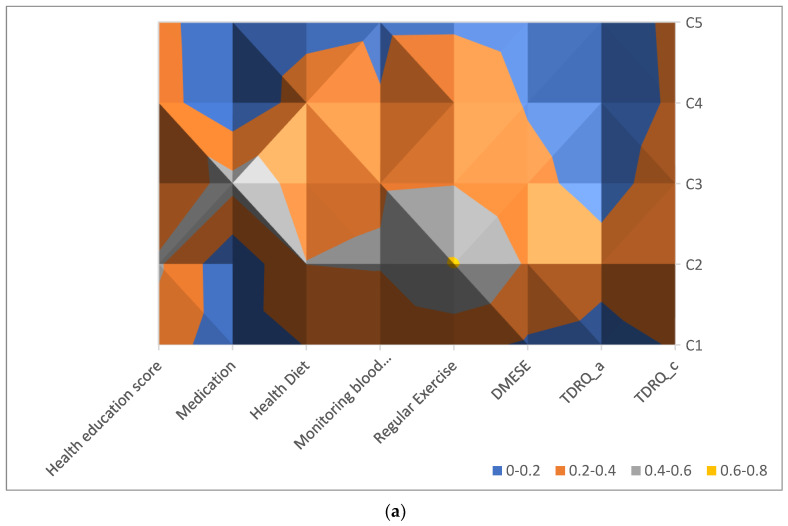

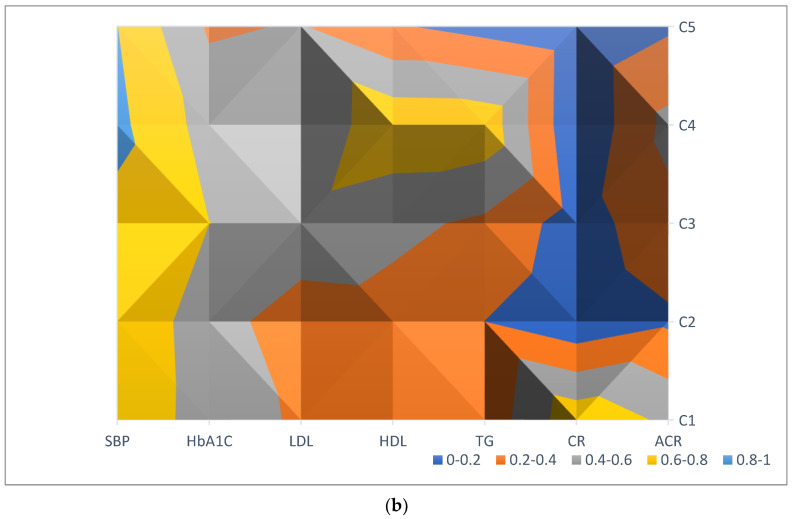
Surface chart of the percentile of indicators from the response frontier for the survey items and normal biomarker levels among the groups. (**a**) Distribution of the participants’ self-management assessment results from the survey. (**b**) The participants’ normal biomarker levels (2013–2019) obtained from the health information system of a hospital.

LPA is a mixed model used for examining unobserved categorical variables by dividing populations into different exclusive latent classes [23]. LPA has been used widely in social sciences [24] and the medical field to cluster individuals into subgroups and to unveil hidden association patterns. After comparing the Akaike information criterion, Bayesian information criterion, and entropy at three, four, and five class levels, we decided to use the five classes from the LPA model. In Table 1, the information from LPA is displayed in five classes, including the information criteria. The proportions of the five categories were 9.8%, 8.2%, 5.8%, 16.3%, and 58.9%. In this patient health profile, the seven biomarkers with normal rate values and self-management assessments were divided into five groups (C1–C5). In addition, Figure 1 presents the distributions of the percentiles of the indicators from the response frontier for the different items. The figure visualizes the five integration types identified from the LCA model. The response frontier is used to calculate the deviation from the highest score for every item due to the wider variations in the item scale ranges. For example, the health education scores for groups C1 and C2 are 6.759 and 5.74, respectively; accordingly, the percentiles of these scores from the response frontier are (10 − 6.759)/10 = 0.32 and (10 − 5.74)/10 = 0.43, respectively.

Figure 1a presents the percentile of every indicator from the response frontier. The items for group C5, for example (presented in blue; 0–0.2), had the smallest percentile from the response frontier, indicating that these participants reported the highest scores for most of the items. The items for group C2 (presented in brown; 0.6–0.8) had the largest percentile from the response frontier, indicating that this group reported the lowest scores for most of the items.

In Figure 1b, the CR and ACR levels in group C1 (presented in yellow; 0.6–0.8) exhibited the largest percentile deviations from the highest normal biomarker levels (=1), whereas the CR and ACR levels for the other groups were small (blue, 0–0.2). The levels in groups C3 (presented in yellow; 0.6–0.8) and C4 (presented in gray; 0.4–0.6) had moderate percentile deviation from the normal biomarker levels.

DMSES: diabetes management self-efficacy scale; TSRQd-A: Treatment Self-Regulation Questionnaire-Diabetes autonomous regulatory style; TSRQd-C: Treatment Self-Regulation Questionnaire-Diabetes controlled regulatory style; SBP: systolic blood pressure; LDL: low-density lipoprotein; HDL: high-density lipoprotein; TG: triglyceride; Cr: creatinine; ACR: albumin creatinine ratio; LPA: latent profile analysis.

### 2.6. Statistical Analyses

We first displayed the descriptions of the participants’ demographic data and the distributions of their responses in the survey items and LPA subgroups among all the participants and divided them according to retinopathy events using the chi-squared test and one-way analysis of variance. Finally, we used a multivariable logistic regression model with determining factors to estimate the risk of retinopathy events. Mplus 7.0 (Muthén and Muthén, Los Angeles, CA, USA), SAS 9.3.1 (SAS Institute, Cary, NC, USA), and SPSS 20.0 (SPSS Inc., Chicago, IL, USA) were used to perform all statistical analyses. The significance level was set at 0.05.

## 3. Results

### 3.1. Basic Characteristics

In this survey, 570 participants were included, and approximately 60% of the participants were male, with a mean age of 62 years and a mean diabetes duration of 11 years (diabetes-years), as shown in Table 2. The mean comprehensive level of health education was 7 (with a maximum score of 10) from the self-reported assessments. The self-assessment scores for diabetes management in the four dimensions (medication, healthy diet, blood glucose monitoring, and regular exercise) were 4.7, 3.94, 3.92, and 3.7, respectively (with a maximum score of 5). Only 16% of the patients felt worse regarding their health status compared with the previous 12 months, while 31% felt better. For the survey items on the DMSES and TSRQd (including autonomous and control regulatory styles), the mean scores were 46, 35, and 26, respectively (with maximum scores of 55, 40, and 35, respectively). In the average biomarker normal rate, the highest was for creatinine (89%), followed by triglyceride (73%) and ACR (73%), while the lowest was for SBP (19%).

The total remaining patients were as follows: 56 patients in C1, 47 in C2, 33 in C3, 93 in C4, and 341 in C5. Furthermore, when we compared the distribution between the two groups (retinopathy events), the TSRQd-A score, SBP, HbA1c, ACR, and LPA in the five classes showed significant differences with regard to age and diabetes duration. Those without DR had higher TSROd-A scores (35 vs. 34, *p* < 0.001), more normal rates of SBP (21% vs. 16%, *p* = 0.002), more normal rates of HbA1c (61% vs. 49%, *p* < 0.001), and more normal rates of ACR (76% vs. 61%, *p* = 0.024) than those with retinopathy.

### 3.2. Multivariable Logistic Regression Model

Finally, we evaluated the factors associated with retinopathy using a multivariable logistic regression model, as shown in Table 3. Those patients who were in the C1, C2, and C4 groups had a higher likelihood of retinopathy events than those who were in the C5 group, with ORs of 1.655 (95% confidence interval (CI): 0.889–3.078, *p* = 0.112), 2.168 (95% CI: 1.093–4.302, *p* = 0.027), and 1.788 (95% CI: 1.058–3.022, *p* = 0.030), respectively. In addition, the risk of retinopathy events increased in those patients who were older (OR = 1.028, 95% CI: 1.008–1.047, *p* = 0.005), whereas it increased in patients with a longer diabetes duration (OR = 1.030, 95% CI: 1.004–1.056, *p* = 0.025). None of the other variables were statistically significant.

## 4. Discussion

We used a novel method that not only considered the patient behaviors during the control of diabetes but also that transformed certain biomarkers into normal rates to catalog the patient health profile subgroups from LPA. Different kinds of patient behavior and biomarkers were used to construct the five groups of patient health profiles, and our findings showed that the use of optimal patient health profiles and having strong patient motivation in diabetes care were associated with better outcomes and could reduce the risk of DR events.

Group C5 had higher scores in the self-management surveys and had the best levels of biomarker normal rates, which were used as the reference; however, those patients who had optimal levels of biomarker normal rates but worse self-reported scores regarding self-management who were placed in group C2 may have an increased risk of retinopathy (OR: 2.168). In addition, those who only have lower scores in health diet, regular exercise, and DMESE-C based on the surveys and an inferior biomarker normal rate in the major items in group C4 and also those patients who have better self-management scores with a normal rate for biomarkers in most of the items but are worse regarding their creatinine and albumin-to-creatinine ratio in group C1 may have an increased risk of retinopathy with ORs of 1.655 and 1.788, respectively. However, those who had worse scores in self-management and biomarker normal rates in the major items in group C3 only had a decreased but not significant risk of retinopathy (OR: 0.689, *p* = 0.466). The possible explanation is that the fewer participants who were assigned to this group from the LPA impaired the functioning fit indices; therefore, a larger sample size and further analyses are needed to provide an adequate statistical power to detect the effect.

Our study found that T2DM patients engaged in healthy behavior, beginning a treatment regimen or who are adhering to a treatment regimen in diabetes care at an optimal level have a decreased likelihood of developing complications in the future. Using biomarkers along with self-management evaluations in constructing patient health profiles can be an efficient strategy to help physicians quickly recognize a high-risk patient group in diabetes care to improve health outcomes in the future by providing individualized health education material.

From the survey, those patients who reported a higher level in survey items (C5), including the self-reported assessment of diabetes care in the four major dimensions (medication, health diet, regular exercise, and monitoring blood glucose) and in health education and self-efficiency scale scores, may have a reduced risk of retinopathy. More disease-related knowledge that is devoted to self-management behavior among patients can help improve the prognosis and outcomes [25,26], especially in diabetic patients, and healthy behavior can help participants maintain both a better lifestyle and a more optimal long-term blood glucose control [27,28,29]. For example, obese patients can reduce their body weight by 5–10%, which can reduce their risk of complications of cardiovascular diseases in the future [30]. Large-scale diabetes control research projects, such as those exhibited in diabetes control and complication trials, have also confirmed the importance of self-management, including self-efficacy, in diabetes control [31,32,33], and this self-management can reduce the risk of complications. In addition, self-management behavior not only involves self-efficiency but is also driven by self-motivation theory, which has been shown to allow patients to develop better self-regulation motivation and is considered to have the ability to help patients achieve their treatment goals and improved blood glucose control [34]. These observations are consistent with the findings in previous studies [25,26].

This study used normal biomarker rate values to represent diabetes control over a period of 7 years, and a higher proportion of normal biomarker rates usually implies better diabetes control. These biomarkers are designed using blood glucose (HbA1C) levels, lipid profiles (LDL, HDL, and TG), blood pressure (SBP), and kidney function (creatinine and ACR), and most studies are in agreement regarding the risk of complications in retinopathy [35]. Traditionally, most studies use the original measure values from those biomarkers and have disadvantages due to having too many items included in the model but also due to treating figure fluctuation as a linear trend, which needs to consider the effect from the regression to the mean [36]. In this study, the use of biomarkers’ normal rates to define long-term diabetes control is a novel approach. In addition, the five groups from the LPA can provide a big picture from either self-management or biomarker dimensions to inspect the outcomes from the concept of patient health profiles. This information can encourage patients and providers to use incentives to improve different patient behaviors in different groups. In further studies, we can apply LPA to a large database to create patient biomarker profiles (or patient health profiles) that can help physicians assess the patient behavior in diabetes care and to develop customized diabetes control plans.

In our study, the incidence of retinopathy was 15.6%, which was lower than that found in other studies [37,38], implying that the subjects in this survey might be healthier or have better control of their T2DM. Most studies agree that a positive relationship exists between severe diabetes and blood glucose levels, and the other apparent factors related to DR are the diabetes duration, poor disease management, and an increasing prevalence over the years [39,40]. The ADA has recommended regular eye examinations as a cost-effective screening strategy [41]. Approximately 60% of patients with T2DM will develop retinopathy within two decades later, and retinopathy can lead to vision loss [39]. These situations require patients to work with a clinical team to reduce the risk. In this study, the OR of diabetes duration was 1.03 after adjustment, which was consistent with previous studies (OR: 1.1–9.0) [42,43,44]. The smaller OR found in this study exists because approximately 90% of the patients with T2DM in the metabolism department of the study hospital received annual eye examinations, as noted in the medical records. By following the ADA recommendations in diabetes care, medical teams may reduce the risk of DR or delay the time of DR occurrence.

For better understanding, we used the seven biomarkers with normal rate values as a traditional variable-oriented method in the multivariable logistic regression model (Supplement file, Table S3). Improved HbA1c levels and ACR may reduce the risk of DR, with ORs of 0.18 and 0.30, respectively. Other studies have also indicated similar findings [45,46]. In addition, the other significant variable in this study is age. Therefore, the results from LPA are appropriate for representing patient behavior in diabetes control, and the statistics were quite stable. Apparently, those patients who were unable to manage their kidney function might have an increased risk of DR, which is in line with the findings of other studies [47,48].

Typically, in previous studies, only one biomarker was used as the factor for disease prediction. This study used seven biomarkers with normal response rates to reflect the other diabetes control dimensions, and these biomarkers could be suitable as long-term indicators. Furthermore, we constructed seven biomarkers with a self-management assessment in the patient health profile from LPA to provide an opportunity for further research and to evaluate the patient behavioral intentions, and this method could be applied to evaluate various complications. Understanding patient behavior is vital to improve the quality and health outcomes in diabetes care.

Some major limitations of this study must be addressed. First, our results were obtained using data from a single center and may not be generalizable to other diabetic patients, even though we used demographic data that did not have an abnormal distribution in the study group. Second, the patients in the hospital voluntarily participated in the survey with written consent; thus, we need to consider the possibility of selection and recall biases, which could affect the results. These participants may have more health awareness in their disease situation; thus, these patients may have more positive factors in their diabetes management. Third, the cross-sectional survey was limited to establishing a temporal association between the exposure and disease conditions. As diabetes care is long-term work, one survey can only reflect the information from the patient’s overall diabetes management at that time point, and this information may be insufficient for evaluating certain specific situations or the long-term aspect of the patient’s diabetes care. Furthermore, we also need to consider information bias, that is, whether some patients believed that their responses might affect their physician’s attitude; thus, these patients may have distorted their answers accordingly. For example, a participant might have answered questions in a manner that would present “a good patient” image, even though the study group confirmed that their providers would not have access to any information relative to the survey responses prior to the study initiation. Fourth, LPA is a novel method, and we provided the selection criteria. We believe that developing biomarker profiles (or health profiles combined with patient behavior evaluations) would be a useful tool to improve the quality and health outcomes of diabetes care; however, we cannot ignore the fact that the classification of biomarker profiles might not currently be suitable for clinical application. Fifth, although we defined DR events from the HIS, DR was diagnosed by physicians, and some patients may have been examined by physicians from the department of endocrinology and metabolism, without being referred to an ophthalmologist. The possibility for undercoding should be a concern, through which some patients with minor disease may not have been diagnosed and coded appropriately in the HIS; thus leading to misclassification bias. Finally, previous studies have mentioned the potential threats that cognitive impairment led to problems with self-management with activities in adherence to the recommendations and glycemic control [49,50,51]. The vulnerable group (especially the elderly group) may be particularly at risk from the negative effects on self-management brought about by cognitive impairment [52]. Further studies may consider the assessment of cognitive function as necessary in prediction models and help patient education intervention to improve self-care behaviors.

## 5. Conclusions

Patient health profiles portraying the patient’s work in diabetes control in the long term can help clinical practitioners categorize the patients with T2DM into different behavior groups. Customizing diabetes care information based on different biomarker profiles may help patients pay more attention to their diabetes control strategies. Designing strategies that can improve patient motivation, either from health authorities or providers, may help patients become more engaged in their diabetes control. Both efforts contribute to reducing the risk of complications; thus leading to better diabetes-related health outcomes in patients and health systems.

## Figures and Tables

**Table 1:** Health profiles from LPA in the circulation indicators with information criteria.

	3 Classes		4 Classes		5 Classes	
AIC	19,851.923		19,625.590		19,568.176	
BIC	20,121.353		19,964.550		19,976.666	
Adjusted BIC	19,924.530		19,716.935		19,678.258	
Entropy	0.844		0.854		0.865	
	Mean	C1	C2	C3	C4	C5
Proportion		9.8%	8.2%	5.8%	16.3%	59.8%
Health education score	7.111	6.759	5.740	**7.440**	**7.250**	**7.323**
Medication	4.654	**4.724**	**4.766**	2.680	**4.738**	**4.804**
Health diet	3.942	**3.955**	2.992	3.181	3.605	**4.257**
Monitoring blood sugar	3.920	**4.000**	2.908	3.112	**3.964**	**4.121**
Regular exercise	3.687	**3.671**	1.902	3.030	3.380	**4.111**
DMESE	45.604	45.379	34.186	41.456	44.712	**47.963**
TSRQd_a	34.848	**34.989**	29.379	34.507	**35.760**	**35.376**
TSRQd_c	26.485	**27.442**	21.813	25.186	**27.189**	**26.904**
SBP	0.194	**0.211**	**0.204**	**0.252**	0.151	**0.197**
HbA1C	0.565	0.509	0.525	0.398	0.480	**0.625**
LDL	0.594	**0.624**	**0.693**	0.473	0.580	0.588
HDL	0.650	0.638	**0.674**	0.551	0.252	**0.780**
TG	0.730	0.739	**0.801**	0.636	0.262	**0.872**
CR	0.888	0.262	**0.957**	**0.898**	**0.980**	**0.972**
ACR	0.724	0.436	**0.833**	0.662	0.543	**0.828**

AIC: Akaike’s information criterion; BIC: Bayesian information criterion; LPA: latent profile analysis; DMSES: diabetes management self-efficacy scale; TSRQd-A: Treatment Self-Regulation Questionnaire-Diabetes autonomous regulatory style; TSRQd-C: Treatment Self-Regulation Questionnaire-Diabetes controlled regulatory style; SBP: systolic blood pressure; LDL: low-density lipoprotein; HDL: high-density lipoprotein; TG: triglyceride; Cr: creatinine; ACR: albumin creatinine ratio.

**Table 2:** The basic characteristics of subjects and items response distribution between whether retinopathy events occurred (*n* = 570).

		Total		Retinopathy None		Retinopathy Positive		*p*
		*n*	%	*n*	%	*n*	%	
		Mean	±SD	Mean	±SD	Mean	±SD	
Sex	Female	226	39.6	162	40.3	64	38.1	0.64
	Male	344	60.4	240	59.7	104	51.9	
Age (mean ± SD)		61.6	12.7	60.0	12.7	65.4	12.0	<0.001 *
Education	Primary school	114	20.0	66	16.4	48	28.6	0.002 *
	Junior High	74	13.0	48	11.9	26	15.5	
	Senior High	161	28.2	117	29.1	44	26.2	
	College (above)	251	3838	171	42.5	50	29.8	
Health status *	Worse	91	16.0	61	30.9	30	17.9	0.731
	Neutral	303	53.3	216	53.9	87	51.8	
	Better	175	30.8	124	15.2	51	30.4	
Health education score		7.12	1.92	7.25	1.81	6.82	2.15	0.018
Medication		4.66	0.67	4.64	0.71	4.70	0.53	0.338
Health diet		3.94	1.04	3.91	0.99	4.02	1.15	0.218
Monitoring blood sugar		3.92	1.39	3.94	1.38	3.87	1.43	0.575
Regular exercise		3.69	1.34	3.66	1.34	3.75	1.34	0.428
Diabetes duration		11.43	7.70	10.61	7.35	13.37	8.16	<0.001 *
DMSES (mean ± SD)		45.59	7.06	45.84	6.70	45.00	7.83	0.200
TSRQd-A (mean ± SD)		34.85	4.21	35.10	4.01	34.26	4.61	0.031 *
TSRQd-C (mean ± SD)		26.49	5.51	26.74	5.44	25.87	5.65	0.088
SBP		0.19	0.15	0.21	0.16	0.16	0.14	0.002 *
HbA1C		0.57	0.34	0.61	0.33	0.49	0.34	<0.001 *
LDL		0.59	0.34	0.59	0.34	0.60	0.35	0.758
HDL		0.65	0.38	0.66	0.38	0.64	0.38	0.585
TG		0.73	0.31	0.74	0.30	0.72	0.32	0.387
CR		0.89	0.25	0.90	0.23	0.86	0.27	0.110
ACR		0.73	0.40	0.76	0.36	0.61	0.45	<0.001 *
LPA	C1	56	9.8	33	58.9	23	41.1	0.024 *
	C2	47	8.2	29	61.7	18	38.3	
	C3	33	5.8	28	84.8	5	15.2	
	C4	93	16.3	61	65.6	32	34.4	
	C5	341	59.8	251	73.6	90	26.4	

Chi-square and ANOVA tests; * comparing with previous 12 months; DMSES: diabetes management self-efficacy scale; TSRQd-A: Treatment Self-Regulation Questionnaire-Diabetes autonomous regulatory style; TSRQd-C: Treatment Self-Regulation Questionnaire-Diabetes controlled regulatory style; SBP: systolic blood pressure; LDL: low-density lipoprotein; HDL: high-density lipoprotein; TG: triglyceride; Cr: creatinine; ACR: albumin creatinine ratio; LPA: latent profile analysis.

**Table 3:** The likelihood of diabetic retinopathy event from multivariable logistic regression model.

		OR	95% UP OR	95% LO OR	*p*
Sex	Male	1.189	0.794	1.780	0.400
Age		1.028	1.008	1.047	0.005 *
Education	Primary school				0.200
	Junior High	0.922	0.480	1.772	0.808
	Senior High	0.694	0.393	1.225	0.208
	College (above)	0.568	0.321	1.005	0.052
Health status *	Worse				0.843
	Neutral	1.050	0.584	1.889	0.871
	Better	0.925	0.539	1.589	0.778
Diabetes duration		1.030	1.004	1.056	0.025 *
LPA	C5				0.032 *
	C1	1.655	0.889	3.078	0.112
	C2	2.168	1.093	4.302	0.027
	C3	0.689	0.252	1.878	0.466
	C4	1.788	1.058	3.022	0.030

OR: odds ratio; * comparing with previous 12 months; DMSES: diabetes management self-efficacy scale; TSRQd-A: Treatment Self-Regulation Questionnaire-Diabetes autonomous regulatory style; TSRQd-C: Treatment Self-Regulation Questionnaire-Diabetes controlled regulatory style; LPA: latent profile analysis.

## Data Availability

The study data are saved on the Survey Research Data Archive (SRDA) in Academia Sinica in Taiwan without health information from the Hospital. Only accessible by approved member and will be archived as per SRDA regulations and national laws. Further information on how to access these data and use are available here: https://srda.sinica.edu.tw/login_y.php (Accessed on 1 August 2021).

## Supplementary Materials

The following supporting information can be downloaded at: https://www.mdpi.com/article/10.3390/ijerph19106084/s1, Table S1: The factor loading from confirmatory factor analysis in c-DMSES items; Table S2: The factor loading from confirmatory factor analysis in TSRQd items; Table S3: The risk of diabetic retinopathy from multivariable logistic regression model.
