# Supplementary material for: Using Patient Health Profile Evaluation for Predicting the Likelihood of Retinopathy in Patients with Type 2 Diabetes: A Cross-Sectional Study Using Latent Profile Analysis
Source: Int J Environ Res Public Health. 2022 May 17;19(10):6084. doi: 10.3390/ijerph19106084 (PMC9141098; doi:10.3390/ijerph19106084)
Supplement: Supplementary file 1 [file ijerph-19-06084-s001.zip › ijerph-1706062-supplementary.pdf]

Using patient health profile evaluation for predicting the likelihood of retinopathy in patients with type 2 diabetes

Shang-Jyh Chiou, Kuomeng Liao, Kuan-Chia Lin, Wender Lin

Table S1 the factor loading from confirmatory factor analysis in c-DMSES items

	Factor 1	Factor 2	Factor 3	Factor 4
c-DMSES 1				.867
c-DMSES 2				.774
c-DMSES 3		.867		
c-DMSES 4		.888		
c-DMSES 5		.597		
c-DMSES 6	.849			
c-DMSES 7	.889			
c-DMSES 8	.776			
c-DMSES 9			.673	
c-DMSES 10			.779	
c-DMSES 11			.749	

c-DMSES items (1-11)

1. I am able to check my blood sugar if necessary
2. I am able to correct my blood sugar when the sugar level is too high (e.g. eat different foods)
3. I am able to do enough physical activity (e.g. walking the dog, yoga, gardening, stretching exercises)
4. I am able to do more physical activity if the doctor advises me to do
5. When doing more physical activity, I am able to adjust my eating plan
6. I am able to follow a healthy eating plan when I am away from home
7. I am able to choose different foods and maintain a healthy eating plan when I am eating out or at a party
8. I am able to maintain my eating plan when I am feeling stressed or anxious
9. I am able to maintain my medication when I am ill
10. I am able to take my medication as prescribed
11. I am able to visit my doctor four times a year to monitor my diabetes

DMSES: diabetes management self-efficacy scale
Principal components analysis
Kaiser-Meyer-Olkin measure of sampling adequacy: 0.789
Bartlett's test, $p < 0.001$

Table S2 the factor loading from confirmatory factor analysis in *TSRQd* items

	Factor 1	Factor 2	Factor 3	Factor 4
TSRQd-1		.622		
TSRQd-2		.826		
TSRQd-3		.826		
TSRQd-4				.772
TSRQd-5	.453		.549	
TSRQd-6			.806	
TSRQd-7			.615	
TSRQd-8	.566		.527	
TSRQd-9		.663		
TSRQd-10	.765			
TSRQd-11	.771			
TSRQd-12				.578
TSRQd-13	.846			
TSRQd-14	.754			
TSRQd-15				.753

TSRQd items (1-15)

1. Other people would be mad at me if I didn't.
2. I would feel bad about myself if I didn't.
3. I would feel guilty if I didn't do what my doctor said.
4. I find it a personal challenge to do so.
5. I personally believe that controlling my diabetes will improve my health.
6. It's exciting to try to keep my glucose in a healthy range.
7. I want my doctor to think I'm a good patient.
8. I personally believe that these are important in remaining healthy.
9. I would be ashamed of myself if I didn't.
10. I've carefully thought about my diet and exercising and believe it's the right thing to do.
11. I feel personally that watching my diet and exercising are the best things for me.
12. I just do it because my doctor said to.
13. I want others to see that I can follow my diet and stay fit.
14. Exercising regularly and following my diet are choices I really want to make.
15. It's a challenge to learn how to live with diabetes.

TSRQd: Treatment Self-Regulation Questionnaire-Diabetes
Principal components analysis
Kaiser-Meyer-Olkin measure of sampling adequacy: 0.831
Bartlett’s test, $p < 0.001$

Table S3 the risk of diabetic retinopathy from multivariable logistic regression model

		OR	95% UP OR	95% LO OR	P
Sex	Male	1.663	0.908	3.045	0.099
Age		1.063	1.027	1.100	<0.001*
Education	Primary school				0.991
	Junior High	1.153	0.465	2.861	0.758
	Senior High	1.063	0.460	2.458	0.886
	College (above)	1.112	0.463	2.673	0.812
Health education score		0.999	0.850	1.175	0.995
Medication		0.843	0.539	1.318	0.454
Health Diet		1.126	0.825	1.537	0.454
Monitoring blood sugar		0.994	0.808	1.223	0.956
Regular Exercise		1.120	0.874	1.436	0.370
Health status*	Worse				0.877
	Neutral	1.247	0.523	2.972	0.618
	Better	1.121	0.505	2.486	0.779
Diabetes duration		0.998	0.958	1.039	0.921
DMSES		0.989	0.937	1.045	0.701
TDRQd-A		0.958	0.884	1.039	0.298
TDRQd-C		1.021	0.965	1.081	0.469
Hba1c		0.182	0.073	0.454	<0.001*
LDL		1.312	0.566	3.042	0.527
HDL		1.101	0.438	2.768	0.838
TG		1.265	0.406	3.945	0.685
SBP		0.055	0.006	0.550	0.014
Creatinine		1.134	0.358	3.596	0.831
ACR		0.302	0.144	0.636	0.002*

OR: odds ratio
*comparing with previous 12 months
DMSES: diabetes management self-efficacy scale
TSRQd-A: Treatment Self-Regulation Questionnaire-Diabetes autonomous regulatory style
TSRQd-C: Treatment Self-Regulation Questionnaire-Diabetes controlled regulatory style
SBP: systolic blood pressure
LDL: low-density lipoprotein
HDL: high-density lipoprotein
TG: Triglyceride
